# Supplementary material for: Integrated Analyses Reveal the Physiological and Molecular Mechanisms of Brassinolide in Modulating Salt Tolerance in Rice
Source: Plants (Basel). 2025 May 21;14(10):1555. doi: 10.3390/plants14101555 (PMC12114769; doi:10.3390/plants14101555)
Supplement: Supplementary file 1 [file plants-14-01555-s001.zip › plants-3606880-supplementary.pdf]

Table S1. RNA-seq profiles of rice leaves after salt stress and BR treatment

Sample	Total Raw Reads (M)	Total Clean Reads (M)	Total Clean Bases(Gb)	Clean Reads Q20(%)	Clean Reads Q30(%)	Total Mapping Genome Ratio(%)	Uniquely Mapping Genome Ratio(%)
C01	47.19	44.63	6.69	97.10	90.81	88.83	86.69
C02	47.19	44.72	6.71	97.22	91.18	90.20	88.02
C03	47.19	44.79	6.72	97.22	91.14	90.54	88.30
C31	47.19	44.83	6.73	97.09	90.75	89.12	87.01
C32	47.19	44.75	6.71	97.02	90.61	89.66	87.54
C33	47.19	44.34	6.65	97.13	90.90	89.65	87.51
CB01	47.19	44.42	6.66	97.34	91.57	90.16	87.94
CB02	47.19	44.40	6.66	97.06	90.70	89.63	87.48
CB03	47.19	44.35	6.65	97.16	91.0	89.98	87.77
CB31	47.19	44.44	6.67	97.27	91.31	89.77	87.67
CB32	47.19	44.69	6.70	97.19	91.08	89.90	87.73
CB33	47.19	45.34	6.80	96.97	90.03	88.93	86.76
H01	47.19	43.99	6.60	96.72	89.94	89.72	87.71
H02	48.93	45.40	6.81	96.85	90.34	89.33	87.14
H03	47.19	44.23	6.63	96.70	89.88	89.82	87.71
H31	47.19	44.54	6.68	96.87	90.36	90.37	88.27
H32	47.19	44.13	6.62	96.72	89.97	88.90	86.62
H33	47.19	43.93	6.59	96.97	90.68	89.83	87.74
HB31	47.19	44.47	6.67	97.20	91.12	89.75	87.58
HB32	47.19	44.57	6.69	97.19	91.09	89.88	87.68
HB33	47.19	44.60	6.69	97.15	90.97	89.63	87.46
HBO1	47.19	44.06	6.61	96.63	89.81	89.72	87.63
HBO2	47.19	44.38	6.66	96.68	89.81	89.82	87.73
HBO3	47.19	44.15	6.62	96.66	89.79	89.59	87.47

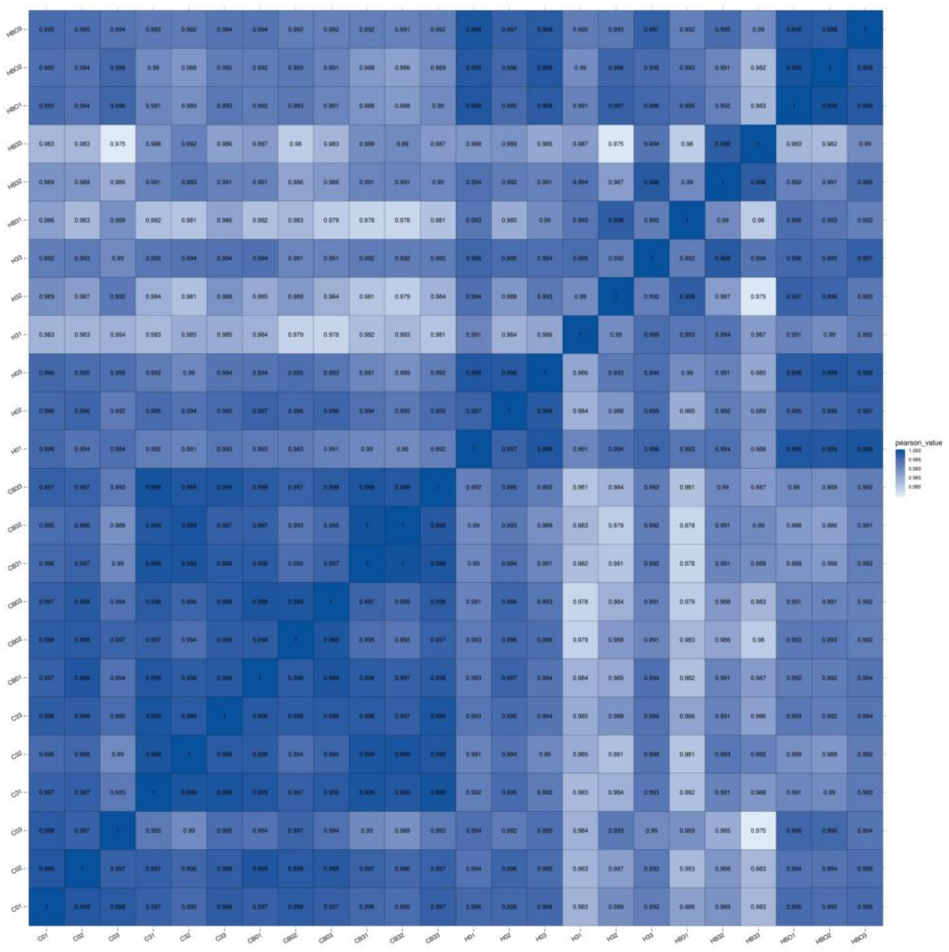

Figure S1. Correlation coefficient between samples

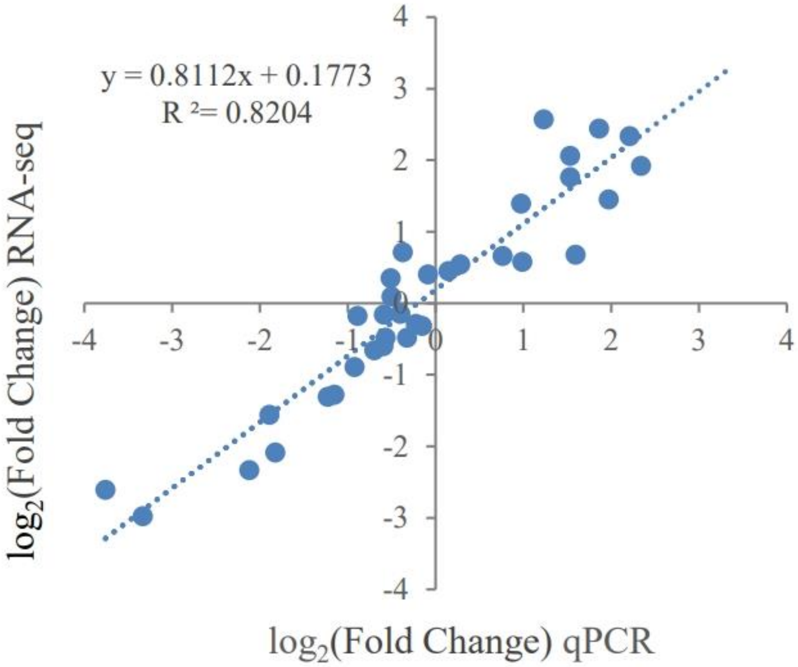

Figure S 2. Correlation analysis revealing the consistency between the RNA sequencing and qRT-PCR data. The RNA sequencing data (x-axis) were plotted against the qRT-PCR data (y-axis).

Table S2. Primers used in qRT-PCR analyses

Functional classification	Gene name	Gene ID	Primer sequences (5' to 3')
Photosynthesis	OsalphaCA3	Os08g0423500-F	CATCCTCTACACCATCGG
		Os08g0423500-R	TCTTTGCCTTCTTGCTTTG
	OsalphaCA4	Os08g0423600-F	GTCATTGGCATCCTCTAC
		Os08g0423600-R	CAACGACCATCTTCTCTT
	OspsbP	Os10g0461400-F	AGGAAGTGGTTGTTCAAG
		Os10g0461400-R	CTGGTTGGAATTGTGTTG
Oxidative stress and defense-response	OsLOX11	Os12g0559200-F	GGACTTCCCTATATTCAG
		Os12g0559200-R	TAATCTGCTCTTCAATCA
	OsLOX12	Os12g0560200-F	AGCATCCAACTTGTCAGGGA
		Os12g0560200-R	TCTGCTGTCATGACCCCATT
	OsJAC1	Os12g0247700-F	GACAGTTCAAGACATCAC
		Os12g0247700-R	CAAGGTAAGT AAACGAGATT
	dihydroflavonol-4-reductase, putative, expressed	Os07g0601900-F	TACAAGGACTTCATCAAC
		Os07g0601900-R	AGATAGTACATCCACCAT
Stress-response	OsWRKY65	Os12g0116800-F	GAAGAGTACTGCTGCTGGGA
		Os12g0116800-R	ACCACCATCAGAACCAGAGG
	OsC3H9	Os01g0645000-F	ACAACCAGGGCATGTTCAAG
		Os01g0645000-R	GGTCTTGTAGCGAGGGTGG
Nitrogen metabolism	OsNADH-GOGAT2	Os05g0555600-F	AGTGGAGGTATTGCTTAT
		Os05g0555600-R	GTGCTGTTGTATCATCAT
Carbon metabolism	OsL85	Os07g0529000-F	AAGGAGTTACTGAAGAGC
		Os07g0529000-R	TGGATTTGGCAAGAACAT
Internal reference gene	ACTIN (nei)-F	Os03g0718100-F	TGAAGTGTGATGTGGATAT
	ACTIN (nei)-R	Os03g0718100-R	CTCATACGGTCAGCAATA

Table S3. Up-regulated or down-regulated differential genes in a comparison of brassinolide + salts and control rice data.

Varieties	Gene ID	symbol	log2FC	P-value
Huanghuazhan	Photosynthesis			
	Os01g0860601	OsFd5	1.28	8.70E-11
	Os07g0147500	OsPsbR1	1.00	0.02
	Os07g0147550	OsPsbR2	1.66	0.02
	Os08g0137800	OsUCL24	-1.94	8.81E-03
	Os04g0678700	OsPORA	1.35	3.26E-06
	Os02g0152400	OsRBCS1	1.55	5.45E-04
	Os08g0423500	OsalphaCA3	-1.28	7.50E-13
	Os06g0726300	ONAC095	-1.08	4.00E-08
	Os03g0133000	ONAC022	-1.96	1.00E-08
	Os07g0683200	OsNAC103	1.25	7.46E-03
	Os01g0862800	ENAC1	-2.11	1.82E-04
	Os11g0154500	OsNAC111	-5.39	1.52E-06
	Os04g0678700	OsPORA	1.35	3.26E-06
	Os09g0397300	OsTPS10	2.09	1.60E-73
	Os08g0445700	OsTPS1	1.88	7.40E-11
	Os09g0376800	OsTPS11	1.67	2.40E-41
	Os05g0517200	OsTPS6	1.29	7.30E-33
	Salt stress response			
	Os08g0448000	Os4CL5	-1.31	1.26E-03
	Os02g0730000	OsALDH2B5	1.38	7.92E-03
	Os10g0529300	OsGSTU7	-1.00	5.00E-09
	Os03g0412800	OsG6PDH4	1.61	6.60E-17
	Os09g0467200	OsGSTU17	-1.17	4.40E-31
	Os04g0180400	CYP99A2	-2.64	1.20E-05
	Os02g0569900	CYP76M7	-3.03	5.49E-06
	Os04g0178300	OsCPS4	-3.94	6.20E-17
	Os02g0755500	UGT85E1	1.10	9.00E-07
	Os02g0115700	OsCAT2	1.28	2.10E-21
	Os12g0435200	OsNCED2	4.04	0.01
	Os03g0100020	OsTET4	1.35	1.39E-04
	Os03g0804500	OsGLP3-7	-1.20	1.02E-05
	Os08g0113000	OsPRX117	-1.11	1.88E-04
	Os12g0569500	OsOSM1	1.12	1.00E-09
	Os01g0975900	OsTIP1; 2	1.13	1.90E-18
	Ion homeostasis			
	Os07g0509800	OsAPRL1	-1.17	9.50E-18
	Os10g0554200	OsNPF6.5	1.06	9.00E-09
	Os08g0403300	HIP46	-1.01	4.21E-03
	Os08g0423500	OsalphaCA3	-1.28	7.50E-13

Os01g0930400	OsHAK5	-1.15	4.23E-06
Os03g0650900	OsNLA2	1.14	1.10E-06
Os02g0306401	OsNAAT1	-1.37	1.90E-19
Os12g0152900	OsbZIP83	-1.56	2.00E-02
Os10g0444700	OsPht1; 8	-1.97	6.70E-17
Os01g0557500	OsCAX1a	-1.19	5.10E-20
Os12g0435200	OsNCED2	4.04	0.01
Os04g0607500	OsHKT1; 1	-1.10	7.40E-11
Os06g0701700	OsHKT2; 1	-1.58	1.40E-10
Os04g0401700	OsHAK1	-2.09	5.10E-13
Os01g0930400	OsHAK5	-1.15	4.23E-06
Lipid metabolism			
Os05g0495700	OsGPDH1-1	1.31	0.02
Os03g0603600	OsGDPD5	-1.13	4.20E-11
Os01g0720400	OsACP1	-1.53	1.70E-41
Os09g0421300	OsPLDα8	1.29	0.03
Os02g0514500	OsGDPD2	-1.32	9.00E-09
Os01g0855000	GPAT	-1.12	0.04
Os08g0508800	OsHI-LOX	-1.66	6.10E-10
Os11g0605500	OsACX2	1.22	2.40E-19
Os08g0509100	OsLOX8	-1.40	1.00E-02
Os03g0225900	OsAOS2	-4.04	6.48E-06
Os02g0730000	OsALDH2B5	1.38	7.92E-03
Os06g0598800	WSL1	-1.02	2.22E-03
Os02g0189200	OsGELP31	-1.44	0.04
Os01g0216400	OsGELP9	-1.57	2.76E-06
Os02g0816200	OsGELP45	-1.37	2.00E-02
Os07g0416900	OsFAD2-3	-3.38	0.02
Hormone			
Os01g0940000	OsCKX4	-2.09	4.5E-46
Os03g0602300	OsDWARF	-1.30	3E-12
Os05g0447200	OsAUX3	1.20	1.07E-05
Os01g0221100	OsJAR2	1.27	2.9E-18
Os02g0574800	OsEIL5	1.27	0.02
Os07g0129300	OsPR1aL	-3.40	3.78E-05
Os01g0382000	OsPR1b	-1.76	0.01
Os01g0846300	OsPP2C09	1.26	9.6E-19
Os11g0143300	OsRR9	-1.13	1.3E-13
Os04g0673300	OsRR6	-1.27	5E-08
Os01g0813100	OsbZIP09	1.69	5.2E-10
Os01g0808100	OsTGA2	1.97	1.9E-21
Os05g0457200	OsPP2C49	1.50	0.01

	Os01g0826400	OsWRKY24	-1.31	2E-08
	Os04g0578000	OsACS2	-1.92	2.1E-12
	Os12g0572000	OsMYB91	-1.31	0.00197
	Os06g0232300	OsPIN1c	-1.33	0.01
	Os05g0482400	EUI1	-1.27	0.01
	Os05g0447200	OsAUX3	1.20	1.07E-05
	Os07g0569100	OsREM4.1	1.01	4.4E-11
	Os05g0514600	OsGA2ox4	-1.46	0.01
	Os05g0500900	OsGH3-4	1.56	0.03
	Os08g0508800	OsHI-LOX	-1.66	6.1E-10
	Os02g0696500	OsXTR3	1.03	0.000298
	Os03g0804500	OsGLP3-7	-1.20	1.02E-05
Chaoyouqianhao	Photosynthesis			
	Os03g0659200	OsFdC1	1.28	1.21E-05
	Os02g0152400	OsRBCS1	1.31	6.33E-03
	Os08g0532200	OsGSAT	1.16	1.10E-11
	Os04g0678700	OsPORA	1.49	1.00E-08
	Os02g0152900	YL1	1.23	4.16E-06
	Os09g0279500	OsCRP7	1.35	7.00E-07
	Os08g0137800	OsUCL24	2.07	9.22E-03
	Os02g0203000	OsbZIP18	-1.02	1.40E-15
	Os03g0236200	OsGAD3	1.88	9.57E-06
	Os12g0564400	OspsbP	1.14	1.71E-03
	Os12g0123800	OsNAC77	3.43	7.12E-04
	Os04g0678700	OsPORA	1.49	1.00E-08
	Os04g0551200	OsMDH4.1	1.58	0.03
	Os02g0121700	OsTPS3	1.09	3.94E-04
	Os02g0790500	OsTPS5	-1.44	1.30E-82
	Salt stress response			
	Os04g0101400	CYP93G1	1.15	4.31E-03
	Os10g0100700	OsPDX1.2	-1.07	1.20E-48
	Os10g0528300	OsGSTU4	3.37	6.33E-03
	Os09g0367700	OsGSTU5	3.47	0.03
	Os10g0527800	OsGSTU12	4.75	0.03
	Os10g0527400	OsGSTU6	7.73	2.07E-03
	Os10g0530900	OsGSTU50	1.60	0.04
	Os03g0412800	OsG6PDH4	1.13	1.00E-09
	Os03g0339300	OsPrx41	1.42	8.00E-03
	Os03g0235000	OsPRX38	1.15	1.58E-03
	Os06g0547400	OsPRX86	1.05	7.20E-18
	Os07g0677500	Osprx114	1.10	3.31E-06

Os03g0368900	OsPRX45	1.55	0.02
Os09g0507500	OsPRX123	3.20	0.02
Os01g0327100	OsPRX15	1.05	0.01
Os05g0135500	OsPRX71	1.10	8.98E-05
Os10g0109600	OsPRX126	1.56	0.01
Os01g0963000	OsPRX22	1.45	4.00E-07
Os02g0115700	OsCAT2	1.27	1.20E-37
Os06g0115400	SOD1-Fe	1.33	7.44E-05
Os04g0551200	OsMDH4.1,	1.58	0.03
Os08g0140300	OsTDC1	3.90	0.05
Os02g0115700	OsCAT2	1.27	1.20E-37
Os04g0101400	CYP93G1	1.15	4.31E-03
Os02g0466400	OsITPK4	6.72	1.85E-03
Os01g0975900	OsTIP1; 2	1.18	1.00E-46
Os09g0541000	OsPIP2	1.31	8.22E-04
Ion homeostasis			
Os03g0196600	OsSAT2; 2	-1.36	2.60E-26
Os01g0720700	OsSAT1; 1	-1.18	1.20E-47
Os03g0100800	OsA8	2.31	0.02
Os02g0649900	OsYSL2	-2.33	2.30E-44
Os07g0258400	OsNRAMP1	-2.74	8.60E-10
Os03g0288000	OsMT1b	1.86	2.00E-08
Os11g0106700	OsFER1	-1.42	3.90E-15
Lipid metabolism			
Os04g0511200	OsClo5	1.83	3.28E-03
Os02g0813600	OsKCS4	1.18	9.00E-08
Os05g0574600	OsCHS	1.15	2.04E-04
Os01g0529800	OsCUT1	1.07	7.16E-03
Os08g0509100	OsLOX8	2.78	7.06E-04
Os04g0445700	OsKAS-IA	1.22	0.04
Os05g0495700	OsGPDH1-1	1.92	6.55E-04
Os05g0448300	GPAT	1.85	0.04
Os02g0514500	OsGDPD2	-1.23	5.00E-08
Os05g0211100	OsCYP51G3	1.07	5.23E-03
Os07g0416900	OsFAD2-3	2.33	0.04
Os08g0509100	OsLOX8	2.78	7.06E-04
Hormone			
Os01g0940000	OsCKX4	-1.19	4.3E-18
Os05g0311801	OsIPT3	4.33	0.02
Os06g0671150	OsSAUR25	1.70	0.03
Os02g0557800	OsRR2	-1.13	6.7E-55
Os01g0924966	OsSAUR3	-3.11	0.03

Os07g0129300	OsPR1aL	2.15	1.9E-12
Os09g0527700	OsIAA26	-3.60	0.03
Os12g0139400	OsRR10	-2.61	1.3E-90
Os03g0268600	OsPP48	-1.04	3E-09
Os02g0226801	OsPYL2	-1.37	0.00428
Os11g0143300	OsRR9	-2.54	6.7E-105
Os04g0673300	OsRR6	-1.62	8.9E-14
Os06g0137400	OsSAUR24	-1.81	0.04
Os01g0952500	OsRR4	-1.03	1.8E-26
Os02g0466400	OsITPK4	6.72	0.00185
Os04g0511200	OsClo5	1.83	0.00328
Os03g0602300	OsDWARF	-1.09	4.06E-05
Os10g0578950	OsACS2	-1.25	1.7E-28
Os03g0227700	OsDWARF4	1.16	0.00201
Os03g0602300	OsDWARF	-1.09	4.06E-05
Os03g0268600	OsPP48	-1.04	3E-09

Table S4. Differential metabolites whose abundance was significantly up-regulated or down-regulated only in brassinolide + salt compared to controls

Varieties	Metabolite ID	Metabolite Name	P-value	VIP	Log_2FC
Huanghuazhan	12.597_550.27653	Convallatoxin	0.00	3.02	-4.89
	12.958_408.24894	3-Methoxy prostaglandin F1α	0.00	2.75	-4.26
	10.241_374.17628	5-Fluoro NNEI	0.00	3.31	-5.82
	14.339_388.22264	1-(3,5-Dihydroxyphenyl)-12-hydroxy-2-tridecanyl acetate	0.00	2.87	-4.24
	14.337_348.23005	β-Cortolone	0.00	2.87	-4.48
	8.769_241.11041	Mefenamic acid	0.00	1.01	-1.28
	13.729_210.12555	Jasmonic acid	0.00	3.55	-7.10
	14.315_366.24061	N-({(1s,4s,6s)-6-isopropyl-3-methyl-4-[2-oxo-2-(1-pyrrolidinyl)ethyl]-2-cyclohexen -1-yl}methyl)-2-py razinecarboxamide	0.00	2.94	-4.52
	13.271_248.17776	Tetranor-12r-hete	0.00	1.57	-1.63
	16.55_363.24095	N-Palmitoyl taurine	0.00	3.22	-5.91
	13.29_266.18797	Tetranor-12r-hete	0.00	1.57	-1.63
	13.924_390.23827	15-Deoxy-Δ12,14-prostaglandinJ2-2-gly cerol ester	0.00	2.96	-4.52
	13.727_326.20920	2,3-dinor prostaglandin e1	0.00	3.14	-5.89
	13.023_369.25153	15-Keto prostaglandin F2α	0.00	3.12	-5.50
	18.97_409.26488	N-benzyl-2-[(3r,4s)-3-{[5-(cyclohexylmet hyl)-1,2-oxazol-3-yl]methyl}-4-piperidin yl]acetamide	0.00	2.43	-2.86
	11.425_204.11533	1-Hydroxycyclohexyl phenyl ketone	0.00	2.47	-3.47
	16.972_344.19630	Nor-9-carboxy-δ9-THC	0.00	2.91	-5.04
	11.068_390.17133	Salvinorin B	0.00	3.46	-5.71
	10.171_374.17629	2-Fluoro NNEI	0.00	3.66	-6.62
	11.83_448.22271	N-({(2R,4S,5R)-5-[3-(4-Fluorophenyl)-1-methyl-1H-pyrazol-5-yl]-1-azabicyclo [2.2.2]oct-2-yl}methyl)-4-methoxybenzam ide	0.00	2.29	-3.03
	13.023_324.19360	15-(tert-Butyl)-2,3,5,6,8,9,11,12-octahydro-1,4,7,10,13-benzopentaoxacyclopentadec ine	0.00	3.19	-5.71
	13.268_316.20380	Cafestol	0.00	1.52	-1.99
	18.682_528.38230	Pachymic acid	0.00	1.91	-2.31
	13.703_367.23586	5-Fluoro JWH 018 adamantyl analog	0.00	2.89	-4.14
	12.957_338.20931	Lauryl gallate	0.00	3.24	-5.82
	16.596_304.24018	(4s,4ar)-4-[(3s)-3-hydroxy-3-methylpent-4-enyl]-3,4a,8,8-tetramethyl-5,6,7,8a-tetr ahydro-4h-napht halen-1-one	0.00	2.72	-3.61

	10.816_441.23743	N-{4-[(1r,9s)-6-oxo-11-(3-phenylpropyl)-7,11-diazatricyclo[7.3.1.02,7]trideca-2,4-dien-3-yl]phenyl}acetamide	0.00	1.80	-2.06
	16.052_380.21755	6,18,19-Trihydroxytrachyloban-2-one	0.00	2.72	-6.24
	10.069_610.26753	Manidipine	0.00	1.92	-2.72
	15.28_292.20359	13(S)-HpOTrE	0.00	2.74	-6.23
	6.627_136.05266	3-Methoxybenzaldehyde	0.00	1.04	0.81
	17.354_290.22442	5α-Dihydrotestosterone	0.00	2.37	-3.57
	11.998_364.22253	Tetrahydrocortisone	0.00	1.44	-1.60
	14.655_362.18596	Remifentanil acid	0.00	1.97	-2.87
	17.857_328.20139	HU-331	0.00	1.54	-1.93
	15.347_218.16730	(+)-Nootkatone	0.00	1.08	-0.78
	17.865_306.21942	16-Heptadecyne-1,2,4-triol	0.00	1.58	-2.01
	15.286_338.20931	Lauryl gallate	0.00	2.81	-6.22
	17.351_330.21699	Testosterone acetate	0.00	1.88	-2.56
	4.255_154.02670	Gentisic acid	0.00	1.17	0.86
	11.369_408.21248	3-{5-[(2s)-1-(cyclohexylmethyl)-2-pyrrolidinyl]-1,2,4-oxadiazol-3-yl}-2-(2-methoxyethoxy)pyridine	0.00	1.62	1.85
	8.349_439.23387	Decoquinate	0.00	2.24	2.74
	8.987_316.05827	Isorhamnetin	0.00	1.37	1.20
	18.954_322.25040	11-Deoxy prostaglandin F1β	0.00	1.74	3.56
	8.967_316.05824	Eupafolin	0.00	1.15	1.02
	16.047_362.20684	Cortisol	0.00	2.28	-4.77
	15.289_383.26728	Myriocin	0.00	2.22	-4.74
	3.999_262.13517	2-(Cyclohexylmethylidene)-1,2,3,4-tetrahydronaphth alen-1-one	0.01	1.09	0.86
	13.111_350.20927	Andrographolide	0.01	1.77	2.21
	9.971_152.04743	2-Hydroxyphenylacetic acid	0.02	4.48	1.32
	14.562_288.20891	Dehydroepiandrosterone (DHEA)	0.02	1.03	-1.34
	16.828_320.23494	8(S)-Hydroxy-(5Z,9E,11Z,14Z)-eicosatetraenoic acid	0.03	1.93	-3.88
	0.864_149.10547	Triethanolamine	0.04	1.72	5.01
	18.822_376.25783	2-(14,15-Epoxyeicosatrienoyl) glycerol	0.01	1.02	0.78
Chaoyouqianhao	16.68_321.26674	α-Linolenoyl ethanolamide	0.00	1.29	-0.45
	5.925_510.19429	Rehmannioside C	0.00	2.42	-1.66
	7.275_524.21025	4-[(β-D-Glucopyranosyloxy)methyl]-6,7-dihydroxy-7-(hydroxymethyl)-1,4a,5,6,7,7a-hexahydrocyclopenta[c]pyran-1-yl3-methylbutanoate	0.00	1.83	-0.93
	6.706_580.14287	Leucoside	0.00	2.02	1.00
	7.78_454.16052	N-{[(2r,4s,5r)-5-{2-methyl-6-[4-(trifluoro	0.00	2.44	1.47

	methyl)phenyl]-4-pyrimidinyl}-1-azabi cyclo[2.2.2]oct-2-yl]methyl}methanesulf onamide			
7.634_462.11592	Homoplantaginin	0.00	1.28	0.77
7.712_162.10467	2,2-Dimethylpropiophenone	0.00	1.43	0.63
10.37_164.12035	5-Phenyl-1-pentanol	0.00	1.49	0.73
6.776_346.21433	Corticosterone	0.00	1.26	-0.63
9.303_404.11080	N1-[3-(Trifluoromethyl)phenyl]-2-[(5-methyl-4-phenyl-1,3-oxazol-2-yl)carbon yl]hydrazine-1-carboxamid e	0.00	1.21	-0.51
13.819_227.26145	Dodecyltrimethylammonium	0.00	2.14	-1.62
6.937_448.15839	Orcinol gentiobioside	0.00	2.51	-1.40
9.186_418.09014	6,7-Dimethoxy-1-methyl-3-isoquinolyl2-methyl-5-ni trobenzene-1-sulfonate	0.00	1.04	-0.43
7.712_190.09962	3-n-Butylphathlide	0.00	1.40	0.63
18.716_425.29322	Jervine	0.00	1.29	-0.55
8.421_374.13668	Diffractic acid	0.00	1.66	-0.54
7.819_406.12657	Astringin	0.00	1.47	0.45
17.841_323.2824	Linoleoyl ethanolamide	0.00	1.57	-0.53
14.181_297.26675	2-Aminooctadec-4-yne-1,3-diol	0.00	1.86	-1.11
10.37_210.12588	Jasmonic acid	0.00	1.25	0.68
7.649_528.18701	1,4:3,6-Dianhydro-2-[(benzylsulfonyl) amino]-5-{[4-(4-biphenylyl)-2-pyrimidi nyl]amino}-2,5-dideoxy-L-iditol	0.00	1.57	0.96
7.621_510.17766	N-(4-{[(1r,9s)-11-(4-fluorobenzyl)-6-oxo-7,11-diazatricyclo[7.3.1.02,7]trideca-2,4-dien-5-yl]sulfamoyl}ph enyl)acetamide	0.00	1.48	1.02
7.712_208.11018	β-Asarone	0.00	1.51	0.68
5.469_226.08430	Sarracenin	0.00	1.08	-0.31
7.829_433.20715	Schisandrol B	0.00	1.74	-0.79
16.347_382.27206	1a,1b-dihomo prostaglandin f2α	0.00	1.46	-0.61
7.012_365.19506	1-[(1s,2s,3s,4r,5r)-4-(diethylamino)-3-hy droxy-6,8-dioxabicyclo[3.2.1]oct-2-yl]-3-(4-methoxyphenyl) urea	0.00	1.38	0.64
6.809_448.10031	Cynaroside	0.00	1.60	1.23
14.268_252.03998	1,3-Dimethyl-4-{[5-(methylthio)-2-thie nyl]methylidene}-4,5-dihydro-1H-pyrazol -5-one	0.00	1.66	1.02
14.265_270.05052	Genistein	0.00	1.68	1.04
17.484_492.24853	2-[(2R,4aS,8S,8aS)-8-{2-[(4aS,7R,8aR)-7-(1-Carboxyvinyl)-1-hydroxy-4a-methyl-2-oxo-1,2,4a,5,6,7,8,8a-octahydro-1-naphth	0.00	1.51	-0.66

	alenyl]ethyl}-4a-methyl-7-oxo-1,2,3,4,4a,7,8,8a-octahydro-2-naphthalenyl]acrylic acid			
13.362_213.24589	1-Tetradecylamine	0.00	1.29	-0.84
21.218_522.35402	Oleic acid-biotin	0.00	1.11	-0.45
12.944_356.19636	Fluanisone	0.00	2.53	2.16
10.205_381.14704	Enrofloxacin	0.00	1.54	-0.86
12.44_332.19846	1,4a-Dimethyl-6-methylene-5-[2-(2-oxo-2,5-dihydro-3-furanyl)ethyl]decahydro-1-naphthalenecarboxylic acid	0.00	2.42	2.91
10.615_496.10022	N-({(2r,4s,5r)-5-[6-(2-bromophenyl)-2-methyl-4-pyrimidinyl]-1-azabicyclo[2.2.2]oct-2-yl}methyl)-2-thiophenecarboxamide	0.00	1.19	-0.35
13.418_302.22458	Abietic acid	0.00	3.58	3.93
10.591_496.10127	N-(2,3-Dihydro-1H-inden-2-yl)-2-[(5-{[2-(2,3-dihydro-1H-inden-2-ylamino)-2-oxoethyl]thio}-1,3,4-thiadiazol-2-yl)thio]acetamide	0.00	1.40	-0.43
15.626_504.24714	N-{[(2r,4s,5s)-5-{[4-(4-fluorophenyl)-1-piperazinyl]methyl}-1-azabicyclo[2.2.2]oct-2-yl]methyl}-4-(trifluoromethyl)benzamide	0.00	1.37	0.73
17.579_348.26625	3-Methyl-5-(5,5,8a-trimethyl-2-methylene-7-oxodecahydro-1-naphthalenyl)pentyl acetate	0.00	1.18	-0.39
1.186_276.09571	N-Hexahydro[1,3]thiazolo[3,4-a]pyridin-3-ylidene-1, 3-benzodioxol-5-amine	0.00	1.13	0.78
12.993_312.22990	(+/-)9-hpode	0.00	1.51	-0.66
7.449_448.10090	Quercetin 7-rhamnoside	0.00	1.26	0.82
7.643_492.12676	Aurantio-obtusin β-D-glucoside	0.00	1.13	0.37
14.48_596.29655	2-{(2S,3R,4S,5R)-5-[(4-{[Benzyl(methyl)amino]methyl}-1H-1,2,3-triazol-1-yl)methyl]-3,4-dihydroxytetrahydro-2-furanyl}-1-[4-(2-methoxyphenyl)-1-piperazinyl]ethanone	0.00	1.71	0.70
13.334_302.18815	trans-D-Allethrin	0.00	3.45	3.09
6.226_300.15728	2-Methyl-5-(2-thienyl)-1-(2,6,6-trimethylcyclohex-2-enyl)penta-1,4-dien-3-one	0.00	1.46	0.71
21.193_256.24002	Palmitic acid	0.00	1.13	-0.51
7.71_226.12067	2-[(3s)-1-methyl-3-pyrrolidinyl]-1h-benzimidazole-5-carbonitrile	0.00	1.49	0.69
12.894_302.22456	Norethandrolone	0.00	2.57	2.24

15.726_482.26409	1-(3-Cyanophenyl)-3-{[(2R,4S,5R)-5-(3-cyclohexyl-1-methyl-1H-pyrazol-5-yl)-1-azabicyclo[2.2.2]oct-2-yl]methyl}urea	0.00	1.37	0.81
13.3_314.18815	Kahweol	0.00	3.83	3.68
7.495_522.13585	5-Hydroxy-2-(4-hydroxy-3-methoxyph enyl)-3,6-dimethoxy-4-oxo-4H-chromen-7-yl β-D-glucopyranoside	0.00	1.15	0.47
15.592_284.21404	(9cis)-Retinal	0.00	2.82	2.69
6.787_448.10059	Cynaroside	0.00	1.76	1.35
19.879_356.29250	Monoolein	0.00	1.29	0.56
20.418_306.25569	11(Z),14(Z),17(Z)-Eicosatrienoic acid	0.00	1.22	-0.61
7.621_492.12705	Aurantio-obtusin β-D-glucoside	0.00	1.40	0.45
12.497_336.23017	16,17-Dihydroxykauran-18-oic acid	0.00	2.20	1.93
7.604_409.20033	N-(2-methoxybenzyl)-2-[(3r,4s)-3-{[5-(met hoxymethyl)-1,2-oxazol-3-yl]methyl}-4-pi peridinyl]acetamide	0.00	1.39	0.70
7.614_462.11635	Diosmetin-7-O-β-D-glucopyranoside	0.00	1.01	0.62
0.893_275.11165	N-Benzyl-N-isopropyl-4-methyl-1,2,3-thiadiazole-5-carboxamide	0.00	1.30	0.68
15.71_300.17201	2-Methoxyestrone	0.00	4.08	3.18
11.314_264.12628	Disperse yellow 39	0.00	3.17	3.48
21.186_282.25567	Trans-petroselinic acid	0.00	1.03	-0.48
15.634_446.24351	1-(4-{5-[(2s)-1-(4-methylbenzyl)-2-pyrroli dinyl]-1,2,4-oxadiazol-3-yl}-2-pyridiny l)-4-piperidinecarboxam ide	0.00	1.29	0.77
13.322_422.22824	(2s,5as,8ar)-6-benzyl-1-methyl-2-[3-(4-morpholinyl)-3-oxopropyl]octahydropy rrolo[3,2-e][1,4]diazepin-5 (2h)-one	0.00	1.26	0.64
5.157_458.27402	1-(3-Cyanophenyl)-3-({(2R,4S,5S)-5-[(4-phenyl-1-piperazinyl)methyl]-1-azabicycl o[2.2.2]oct-2-yl}methy l)urea	0.00	1.34	-0.50
15.347_218.16730	(+)-Nootkatone	0.00	1.64	-0.64
4.778_420.21226	4-Benzyl-2-({4-[5-(trifluoromethyl)-2-pyridinyl]pipe razino}methyl)	0.00	1.49	0.48
14.865_286.22987	Arachidonic acid	0.00	2.87	2.72
4.631_264.14742	Ethyl4-(2-methoxyphenyl)piperazine-1-carboxylate	0.00	1.22	0.58
0.864_149.10547	Triethanolamine	0.00	5.33	7.67
0.897_276.09568	ϒ-L-Glutamyl-L-glutamic acid	0.00	1.45	0.71
0.866_542.12589	Rhusflavanone	0.00	1.28	0.83
18.811_354.2760	1-Linoleoyl glycerol	0.00	1.17	0.51
0.96_313.06754	Triazophos	0.00	1.44	0.67

0.919_106.02654	(2R)-2,3-Dihydroxypropanoic acid	0.00	1.55	0.80
8.219_467.22885	1-{[(1s,4s,6s)-6-isopropyl-3-methyl-4-{[5-(4-pyridinyl)-1,3,4-oxadiazol-2-yl]methyl}-2-cyclohexen-1-yl] methyl}-3-phenylurea	0.00	1.16	0.57
13.268_316.20380	Cafestol	0.01	2.77	2.25
12.944_334.21388	6,18,19-Trihydroxytrachyloban-2-one	0.01	1.04	0.87
6.751_392.20595	5-Fluoro-3,5-AB-PFUPPYCA	0.01	1.45	0.73
1.709_180.06338	D-(-)-Fructose	0.01	2.24	-1.15
8.769_241.11041	Mefenamic acid	0.01	1.58	1.20
8.758_464.12988	Loprazolam	0.01	1.05	0.51
18.835_336.23002	15(s)-hpete	0.01	1.26	0.79
9.971_152.04743	2-Hydroxyphenylacetic acid	0.01	4.48	4.79
5.285_488.24804	N-[(1s,3as,5s,7ar)-7a-{3-[(2-furylmethyl)amino]-3-oxopropyl}-5-hydroxy-3,3,5-trimethyloctahydro-1h-inden-1-yl]benzamide	0.01	1.30	-0.39
6.06_479.23791	N-{[(1s,4s,6s)-4-{2-[(4-cyanobenzyl)amino]-2-oxoethyl}-6-isopropyl-3-methyl-2-cyclohexen-1-yl]methy l}benzamide	0.01	1.01	0.49
0.859_202.14313	N3,N4-Dimethyl-L-arginine	0.01	1.50	-0.56
10.312_267.12596	6,7-Dimethoxy-1-phenyl-3,4-dihydroisoquinoline	0.01	2.27	3.11
3.753_158.08464	1,5-Naphthalenediamine	0.01	1.17	-0.45
13.331_442.22595	1-{[(2r,3s,4r,5s)-3,4-dihydroxy-5-{2-[(2r)-2-(methoxymethyl)-1-pyrrolidinyl]-2-oxoethyl}tetrahydro-2-fur anyl]methyl}-3-(3-fluorophenyl)urea	0.01	2.41	1.57
4.457_262.13177	Methohexital	0.01	1.13	-0.41
18.682_528.38230	Pachymic acid	0.01	1.15	0.77
13.328_382.23557	N-{[(2R,4S,5R)-5-(3-Cyclopentyl-1-methyl-1H-pyrazol-5-yl)-1-azabicyclo[2.2.2]oct-2-yl]methyl}-2-fura mide	0.01	1.20	0.64
5.222_301.14262	(4ar,7as)-6-(cyclopropylcarbonyl)-4-(3-pyridinylmethyl)hexahydropyrrolo[3,4-b][1,4]oxazin-3(2h)-one	0.01	1.31	-0.44
18.83_336.26494	2-Linoleoyl glycerol	0.02	1.58	0.70
11.314_143.07375	2-Naphthylamine	0.02	2.33	2.71
11.83_448.22271	N-({(2R,4S,5R)-5-[3-(4-Fluorophenyl)-1-methyl-1H-pyrazol-5-yl]-1-azabicyclo[2.2.2]oct-2-yl}methyl)-4-methoxybenzamide	0.02	1.53	1.24
6.477_315.12178	N4-Benzoyl-2',3'-dideoxycytidine	0.02	1.10	-0.53

10.74_162.10466	Valerophenone	0.02	1.15	-0.35
8.103_334.15293	N-{4-[(2r,3r)-3-(hydroxymethyl)-5-oxo-2-morpholinyl]phenyl}tetrahydro-2h-pyran -4-carboxamide	0.02	1.37	1.29
5.624_327.21571	Butorphanol	0.02	1.02	-0.44
4.84_261.11107	Imazapyr	0.03	1.02	-0.31
16.857_340.20720	Esculin	0.03	1.30	-0.51
17.584_477.31488	Victoria Pure Blue BO	0.03	2.30	1.02
6.316_483.22320	Methyl(2S,4S,6S,12bR)-2-(benzylamino)-4-(2-fluorophenyl)-1,2,3,4,6,7,12,12b-oct ahydroindolo[2,3-a]quinolizine-6-carboxy late	0.03	1.03	0.53
12.119_286.0840	Sakuranetin	0.03	3.92	7.44
16.242_422.30344	1-[(2R,4S,5R)-5-(3-Cyclohexyl-1-methyl-1H-pyrazol -5-yl)-1-azabicyclo[2.2.2]oct-2-yl]-N-(4-methoxyben zyl)methanamine	0.04	1.21	0.64
9.97_108.05754	4-Methylphenol	0.04	3.23	3.38
0.889_130.02681	L-Glutamic acid	0.04	1.07	0.55
15.709_404.25639	Lovastatin	0.04	1.55	0.81
4.922_305.13751	Imazamox	0.04	1.02	-0.26
1.546_404.19069	2-[(3R,4S)-1-Benzoyl-3-ethyl-4-piperidi nyl]-N-(4-flu orobenzyl)acetamide	0.05	1.48	-0.67

Table S5. KEGG annotation approach of brassinolide + salt treatment of rice metabolites

Varieties	Metabolite ID	Compounds	KEGG annotation	KEGG Pathway annotation
Huanghuazhan	12.597_550.27653	Convallatoxin	--	unknown
	12.958_408.24894	3-Methoxy prostaglandin F1α	--	unknown
	10.241_374.17628	5-Fluoro NNEI	--	unknown
	14.339_388.22264	1-(3,5-Dihydroxyphenyl)-12-hydrox y-2-tridecanyl acetate	--	unknown
	14.337_348.23005	β-Cortolone	--	unknown
	8.769_241.11041	Mefenamic acid	C02168	unknown
	14.315_366.24061	N-({(1s,4s,6s)-6-isopropyl-3-methyl-4-[2-oxo-2-(1-pyrrolidinyl)ethyl]-2-cyclohexen-1-yl}methyl)-2-pyrazineca rboxamide	--	unknown
	13.271_248.17776	Tetranor-12r-hete	--	unknown
	13.729_210.12555	Jasmonic acid	C08491	Aalpha-Linolenic acid metabolism (ko00592)<br>Biosynthesis of plant secondary metabolites(ko01060)<br>Biosynthesis of plant hormones(ko01070)<br>Plant hormone signal transduction(ko04075)
	16.55_363.24095	N-Palmitoyl taurine	--	unknown
	13.29_266.18797	Tetranor-12r-hete	--	unknown
	13.924_390.23827	15-Deoxy-Δ12,14-prostaglandin J2-2-glycerol ester	--	unknown
	13.023_369.25153	15-Keto prostaglandin F2α	C05960	Arachidonic acid metabolism(ko00590)
	13.727_326.20920	2,3-dinor prostaglandine1	--	unknown
	18.97_409.26488	N-benzyl-2-[(3r,4s)-3-{[5-(cyclohexylmethyl)-1,2-oxazol-3yl]m ethyl}-4-piperidinyl]acetamide	--	unknown
	11.425_204.11533	1-Hydroxycyclohexylphenylketone	--	unknown
	16.972_344.19630	Nor-9-carboxy-δ9-THC	--	unknown
	11.068_390.17133	Salvinorin B	--	unknown
	10.171_374.17629	2-Fluoro NNEI	--	unknown
	13.023_324.19360	15-(tert-Butyl)-2,3,5,6,8,9,11,12-octahydro-1,4,7,10,13-benzopentaoxacyclopentadecine	--	unknown
	11.83_448.22271	N-({(2R,4S,5R)-5-[3-(4-Fluorophen	--	unknown

	yl)-1-methyl-1H-pyrazol-5-yl]-1-azabicyclo[2.2.2]oct-2-yl}methyl)-4-met hoxybenzamide		
13.268_316.20380	Cafestol	C09066	unknown
18.682_528.38230	Pachymic acid	C17044	unknown
13.703_367.23586	5-Fluoro JWH 018 adamantyl analog	--	unknown
12.957_338.20931	Lauryl gallate	--	unknown
16.596_304.24018	(4s,4ar)-4-[(3s)-3-hydroxy-3-methylpent-4-enyl]-3,4a,8,8-tetramethyl-5,6,7,8a-tetrahydro-4h-naphthalen-1-one	--	unknown
10.816_441.23743	N-{4-[(1r,9s)-6-oxo-11-(3-phenylpropyl)-7,11-diazatricyclo[7. 3.1.02,7]trideca-2,4-dien-3-yl]phenyl}acetamide	--	unknown
16.052_380.21755	6,18,19-Trihydroxytrachyloban-2-one	--	unknown
10.069_610.26753	Manidipine	D08155	unknown
15.28_292.20359	13(S)-HpOTrE	--	unknown
6.627_136.05266	3-Methoxybenzaldehyde	--	unknown
17.354_290.22442	5α-Dihydrotestosterone	C03917	Steroid hormone biosynthesis(ko00140)
11.998_364.22253	Tetrahydrocortisone	C05470	unknown
14.655_362.18596	Remifentanil acid	--	unknown
17.857_328.20139	HU-331	--	unknown
15.347_218.16730	(+)-Nootkatone	--	unknown
17.865_306.21942	16-Heptadecyne-1,2,4-triol	--	unknown
15.286_338.20931	Lauryl gallate	--	unknown
17.351_330.21699	Testosterone acetate	C03027	unknown
11.369_408.21248	3-{5-[(2s)-1-(cyclohexylmethyl)-2-pyrrolidinyl]-1,2,4-oxadiazol-3-yl}-2-(2 -methoxyethoxy)pyridine	--	unknown
4.255_154.02670	Gentisic acid	C00628	Tyrosine metabolism(ko00350)
8.349_439.23387	Decoquinate	D03667	unknown
8.987_316.05827	Isorhamnetin	C10084	unknown
18.954_322.25040	11-Deoxy prostaglandin F1β	--	unknown
8.967_316.05824	Eupafolin	--	unknown
16.047_362.20684	Cortisol	C00735	Steroid hormone biosynthesis(ko00140)<br>Aldosterone-regulated sodium reabsorption(ko04960)<br>Glucocorticoid and mineralocorticoidreceptor agonists/antagonists(ko07225)

	15.289_383.26728	Myriocin	C19914	unknown
	3.999_262.13517	2-(Cyclohexylmethylidene)-1,2,3,4-tetrahydronaphthalen-1-one	--	unknown
	13.111_350.20927	Andrographolide	C20214	unknown
	9.971_152.04743	2-Hydroxyphenylacetic acid	C05852	Phenylalanine metabolism(ko00360) Styrene degradation(ko00643)
	14.562_288.20891	Dehydroepiandrosterone (DHEA)	C01227	Steroid hormone biosynthesis(ko00140) Steroid degradation(ko00984)
	16.828_320.23494	8(S)-Hydroxy-(5Z,9E,11Z,14Z)-eicos atetraenoic acid	C14776	Arachidonic acid metabolism(ko00590)
	0.864_149.10547	Triethanolamine	C06771	Glycerophospholipid metabolism(ko00564)
	18.822_376.25783	2-(14,15-Epoxyeicosatrienoyl)glycerol	--	unknown
Chaoyouqianhao	16.68_321.26674	α-Linolenoyl ethanolamide	--	unknown
	5.925_510.19429	Rehmannioside C	--	unknown
	7.275_524.21025	4-[(β-D-Glucopyranosyloxy)methyl]-6,7-dihydroxy-7-(hydroxymethyl)-1,4a,5,6,7,7a-hexahydrocyclopenta[c]pyran-1-yl3-methylbutanoate	--	unknown
	6.706_580.14287	Leucoside	--	unknown
	7.78_454.16052	N-{[(2r,4s,5r)-5-{2-methyl-6-[4-(trif luoromethyl)phenyl]-4-pyrimidin yl}-1-azabicyclo[2.2.2]oct-2-yl]met hyl}methanesulfonamide	--	unknown
	7.634_462.11592	Homoplantaginin	C17762	unknown
	6.776_346.21433	Corticosterone	C02140	Steroid hormone biosynthesis(ko00140)
	7.712_162.10467	2,2-Dimethylpropiophenone	--	unknown
	10.37_164.12035	5-Phenyl-1-pentanol	--	unknown
	9.303_404.11080	N1-[3-(Trifluoromethyl)phenyl]-2-[(5 -methyl-4-phenyl-1,3-oxazol-2-yl)carbonyl]hydrazine-1-carboxamide	--	unknown
	13.819_227.26145	Dodecyltrimethylammonium	C20389	unknown
	7.819_406.12657	Astringin	C10245	unknown
	6.937_448.15839	Orcinol gentiobioside	--	unknown
	9.186_418.09014	6,7-Dimethoxy-1-methyl-3-isoquinolyl2-methyl-5-nitrobenzene-1-	--	unknown

	sulfonate		
18.716_425.29322	Jervine	C10811	Biosynthesis of alkaloids derived from terpenoid and polyketide(ko01066)
7.712_190.09962	3-n-Butylphathlide	C17854	unknown
8.421_374.13668	Diffractic acid	--	unknown
17.841_323.2824	Linoleoyl ethanolamide	--	unknown
14.181_297.26675	2-Aminooctadec-4-yne-1,3-diol	--	unknown
10.37_210.12588	Jasmonic acid	C08491	Alpha-Linolenic acid metabolism(ko0059 2)<br>Biosynthesis of plant hormones(ko01070)<br>Plant hormone signal transduction(ko04 075)<br>Biosynthesis of plant secondary metabo lites(ko01060)
7.649_528.18701	1,4:3,6-Dianhydro-2-[(benzylsulfo nyl)amino]-5-{[4-(4-biphenylyl)-2-pyri midinyl]amino}-2,5-dideoxy-L-iditol	--	unknown
5.469_226.08430	Sarracenin	--	unknown
6.809_448.10031	Cynaroside	C03951	Flavone and flavonol biosynthesis (ko009 44)
7.829_433.20715	Schisandrol B	--	unknown
7.621_510.17766	N-(4-{[(1r,9s)-11-(4-fluorobenzyl)-6-oxo-7,11-diazatricyclo[7.3.1.02, 7]trideca-2,4-dien-5yl]sulfamoyl}p henyl)a cetamide	--	unknown
7.712_208.11018	β-Asarone	C10430	unknown
16.347_382.27206	1a,1b-dihomo prostaglandin f2α	--	unknown
17.484_492.24853	2-[(2R,4aS,8S,8aS)-8-{2-[(4aS,7R,8 aR)-7-(1-Carboxyvinyl)-1-hydro xy-4a-methyl-2-oxo-1,2,4a,5, 6,7,8, 8a-octahydro-1-naphthalenyl]eth yl}-4a-methyl-7-oxo-1,2,3,4,4a,7,8, 8a-octahydro-2-naphthalenyl]acr ylic acid	--	unknown
13.362_213.24589	1-Tetradecylamine	--	unknown
7.012_365.19506	1-[(1s,2s,3s,4r,5r)-4-(diethylami no)-3-hydroxy-6,8dioxabicyclo[3. 2.1]oct-2-yl]-3-(4-methoxypheny l)urea	--	unknown
14.268_252.03998	1,3-Dimethyl-4-{[5-(methylthio)-2-thienyl]methylidene}-4,5-dihydro-	--	unknown

	1H-p yrazol-5-one		
14.265_270.05052	Genistein	C06563	Biosynthesis of phenylpropanoids(ko01061)
21.218_522.35402	Oleic acid-biotin	--	unknown
10.205_381.14704	Enrofloxacin	D02473	unknown
12.944_356.19636	Fluanisone	D02621	unknown
10.615_496.10022	N-({(2r,4s,5r)-5-[6-(2-bromophenyl)-2-methyl-4-pyrimidinyl]-1-azabicyclo[2.2.2]oct-2-yl}methyl)-2-thiophenec arboxamide	--	unknown
12.44_332.19846	1,4a-Dimethyl-6-methylene-5-[2-(2-oxo-2,5-dihydro-3-furanyl)ethyl] decahydro-1-naphthalenecarboxylic acid	--	unknown
13.418_302.22458	Abietic acid	C06087	Diterpenoid biosynthesis(ko00904)
10.591_496.10127	N-(2,3-Dihydro-1H-inden-2-yl)-2-[(5-{[2-(2,3-dihydro-1H-inden-2-ylamino)-2-oxoethyl]thio}-1,3,4-thiadiazol-2-yl)thio]acetamide	--	unknown
17.579_348.26625	3-Methyl-5-(5,5,8a-trimethyl-2-methylene-7-oxodecahydro-1-naphthalenyl) pentyl acetate	--	unknown
7.643_492.12676	Aurantio-obtusin β-D-glucoside	C10303	unknown
14.48_596.29655	2-{(2S,3R,4S,5R)-5-[(4-{[Benzyl(methyl)amino]methyl}-1H-1,2,3-triazol-1-yl)methyl]-3,4-dihydroxytetrahydro-2-furanyl}-1-[4-(2-methoxyphenyl)-1-piperazinyl]ethanone	--	unknown
15.626_504.24714	N-{[(2r,4s,5s)-5-{[4-(4-fluorophenyl)-1-piperazinyl]methyl}-1-azabicyclo[2.2.2]oct-2-yl]methyl}-4-(trifluorom ethyl)benzamide	--	unknown
12.993_312.22990	(+/-)9-hpode	--	unknown
21.193_256.24002	Palmitic acid	C00249	Fatty acid biosynthesis(ko00061)<br>Fatty acid elongation(ko00062)<br>Fatty acid degradation(ko00071)<br>Cutin, suberine and wax biosynthesis (ko 00073)<br>Fatty acid metabolism(ko01212)
1.186_276.09571	N-Hexahydro[1,3]thiazolo[3,4-a]pyridin-3-ylidene-1,3-benzodioxol-5-amine	--	unknown

15.726_482.26409	1-(3-Cyanophenyl)-3-{[(2R,4S,5R)-5-(3-cyclohexyl-1-methyl-1H-pyrazol-5-yl)-1-azabicyclo[2.2.2]oct-2-yl]m ethyl}urea	--	unknown
7.449_448.10090	Quercetin 7-rhamnoside	--	unknown
13.334_302.18815	Trans-D-Allethrin	--	unknown
12.497_336.23017	16,17-Dihydroxykauran-18-oic acid	--	unknown
6.226_300.15728	2-Methyl-5-(2-thienyl)-1-(2,6,6-trimethylcyclohex-2-enyl)penta-1,4-dien-3-one	--	unknown
7.71_226.12067	2-[(3s)-1-methyl-3-pyrrolidinyl]-1h-b enzimidazole-5-carbonitrile	--	unknown
12.894_302.22456	Norethandrolone	D07127	unknown
13.3_314.18815	Kahweol	--	unknown
7.495_522.13585	5-Hydroxy-2-(4-hydroxy-3-methoxyphenyl)-3,6-dimethoxy-4-oxo-4H-chromen-7-ylβ-D-glucopyranoside	--	unknown
15.592_284.21404	(9cis)-Retinal	C16681	Retinol metabolism(ko00830)
6.787_448.10059	Cynaroside	C03951	Flavone and flavonol biosynthesis(ko00944)
20.418_306.25569	11(Z),14(Z),17(Z)-Eicosatrienoic acid	C16522	Biosynthesis of unsaturated fatty acids(ko01040)
19.879_356.29250	Monoolein	--	unknown
21.186_282.25567	Trans-petroselinic acid	--	unknown
7.621_492.12705	Aurantio-obtusin β-D-glucoside	C10303	unknown
7.604_409.20033	N-(2-methoxybenzyl)-2-[(3r,4s)-3-{[5-(methoxymethyl)-1,2-oxazol-3-yl]methyl}-4-piperidinyl]acetamide	--	unknown
5.157_458.27402	1-(3-Cyanophenyl)-3-({(2R,4S,5S)-5-[(4-phenyl-1-piperazinyl)methyl]-1-azabicyclo[2.2.2]oct-2-yl}methyl)urea	--	unknown
7.614_462.11635	Diosmetin-7-O-β-D-glucopyranoside	--	unknown
0.893_275.11165	N-Benzyl-N-isopropyl-4-methyl-1,2,3 -thiadiazole-5-carboxamide	--	unknown
15.71_300.17201	2-Methoxyestrone	C05299	Steroid hormone biosynthesis(ko00140)
11.314_264.12628	Disperse yellow 39	--	unknown
15.634_446.24351	1-(4-{5-[(2s)-1-(4-methylbenzyl)-2-	--	unknown

	pyrrolidinyl]-1,2,4-oxadiazol-3-yl}-2-pyridinyl)-4-piperidinecarboxamide		
13.322_422.22824	(2s,5as,8ar)-6-benzyl-1-methyl-2-[3-(4-morpholinyl)-3-oxopropyl]octahydropyrrolo[3,2-e][1,4]diazepin-5(2h)-one	--	unknown
15.347_218.16730	(+)-Nootkatone	--	unknown
4.778_420.21226	4-Benzyl-2-({4-[5-(trifluoromethyl)-2-pyridinyl]piperazino}methyl)	--	unknown
0.919_106.02654	(2R)-2,3-Dihydroxypropanoic acid	C00258	Pentose phosphate pathway(ko00030)<br>Glycine, serine and threonine metabolism(ko00260)<br>Glycerolipid metabolism(ko00561)
14.865_286.22987	Arachidonic acid	C00219	Arachidonic acid metabolism(ko00590)<br>Linoleic acid metabolism(ko00591)<br>Biosynthesis of unsaturated fatty acids (ko01040)
4.631_264.14742	Ethyl4-(2-methoxyphenyl)piperazine-1-carboxylate	--	unknown
6.751_392.20595	5-Fluoro-3,5-AB-PFUPPYCA	--	unknown
0.864_149.10547	Triethanolamine	C06771	Glycerophospholipid metabolism(ko00564)
0.897_276.09568	ϒ-L-Glutamyl-L-glutamic acid	C05282	unknown
1.709_180.06338	D-(-)-Fructose	C02336	Amino sugar and nucleotide sugar metabolism(ko00520)
0.866_542.12589	Rhusflavanone	--	unknown
18.811_354.27609	1-Linoleoyl glycerol	--	unknown
0.96_313.06754	Triazophos	C18657	unknown
8.219_467.22885	1-{[(1s,4s,6s)-6-isopropyl-3-methyl-4-{[5-(4-pyridinyl)-1,3,4-oxadiazol-2-yl]methyl}-2-cyclohexen-1-yl]methyl}-3-phenylurea	--	unknown
13.268_316.20380	Cafestol	C09066	unknown
12.944_334.21388	6,18,19-Trihydroxytrachyloban-2-one	--	unknown
18.835_336.23002	15(s)-hpete	C05966	Arachidonic acid metabolism(ko00590)
9.971_152.04743	2-Hydroxyphenylacetic acid	C05852	Phenylalanine metabolism(ko00360)<br>Styrene degradation(ko00643)
5.285_488.24804	N-[(1s,3as,5s,7ar)-7a-{3-[(2-furylmethyl)amino]-3-oxopropyl}-5-hydr	--	unknown

	oxy-3,3,5-trimethyloctahydro-1h-inden-1-yl] benzamide		
6.06_479.23791	N-{[(1s,4s,6s)-4-{2-[(4-cyanobenzy l) amino]-2-oxoethyl}-6-isopropyl-3-methyl-2-cyclohexen-1-yl]meth yl} benzamide	--	unknown
8.769_241.11041	Mefenamic acid	C02168	unknown
8.758_464.12988	Loprazolam	D07326	unknown
0.859_202.14313	N3,N4-Dimethyl-L-arginine	--	unknown
10.312_267.12596	6,7-Dimethoxy-1-phenyl-3,4-dihydro isoquinoline	--	unknown
3.753_158.08464	1,5-Naphthalenediamine	C19463	unknown
13.331_442.22595	1-{[(2r,3s,4r,5s)-3,4-dihydroxy-5-{2-[(2r)-2-(methoxymethyl)-1-py rrolidin yl]-2-oxoethyl}tetrahydro-2-furanyl]methyl}-3-(3-fluorophe nyl)urea	--	unknown
4.457_262.13177	Methohexital	C07844	unknown
18.682_528.38230	Pachymic acid	C17044	unknown
11.83_448.22271	N-({(2R,4S,5R)-5-[3-(4-Fluorophen yl)-1-methyl-1H-pyrazol-5-yl]-1-azabicyclo[2.2.2]oct-2-yl}methyl)-4-methoxybenzamide	--	unknown
5.222_301.14262	(4ar,7as)-6-(cyclopropylcarbonyl)-4-(3-pyridinylmethyl)hexahydrop yrrolo [3,4-b][1,4]oxazin-3(2h)-one	--	unknown
13.328_382.23557	N-{[(2R,4S,5R)-5-(3-Cyclopentyl-1-methyl-1H-pyrazol-5-yl)-1-azabicyclo[2.2.2]oct-2-yl]methyl}-2-fura mide	--	unknown
6.477_315.12178	N4-Benzoyl-2',3'-dideoxycytidine	--	unknown
18.83_336.26494	2-Linoleoyl glycerol	--	unknown
8.103_334.15293	N-{4-[(2r,3r)-3-(hydroxymethyl)-5-oxo-2-morpholinyl]phenyl}tetra hydro-2h-pyran-4-carboxamide	--	unknown
11.314_143.07375	2-Naphthylamine	C02227	unknown
10.74_162.10466	Valerophenone	--	unknown
5.624_327.21571	Butorphanol	C06863	unknown
4.84_261.11107	Imazapyr	C18864	unknown
6.316_483.22320	Methyl(2S,4S,6S,12bR)-2-(benzyla mIno)-4-(2-fluorophenyl)-1,2,3,4,6,7,12,12b-octahydroindolo[2,3-a]	--	unknown

	quinoli zine-6-carboxylate		
16.857_340.20720	Esculin	--	unknown
9.97_108.05754	4-Methylphenol	C01468	Protein digestion and absorption(ko04974)
17.584_477.31488	Victoria Pure Blue BO	--	unknown
12.119_286.08409	Sakuranetin	--	unknown
16.242_422.30344	1-[(2R,4S,5R)-5-(3-Cyclohexyl-1-methyl-1H-pyrazol-5-yl)-1-azabicyclo[2.2.2]oct-2-yl]-N-(4-methoxybenzyl) methanamine	--	unknown
0.889_130.02681	L-Glutamic acid	C00025	Alanine, aspartate and glutamate metabolism(ko00250)<br>Taurine and hypotaurine metabolism(ko00430)<br>map Glutathione metabolism(ko00480)<br>Nitrogen metabolism(ko00910)<br>Porphyrin and chlorophyll metabolism (ko00860)<br>Glyoxylate and dicarboxylate metabolism(ko00630)<br>D-Glutamine and D-glutamate metabolism(ko00471)<br>Carbon metabolism(ko01200)
15.709_404.25639	Lovastatin	--	unknown
4.922_305.13751	Imazamox	C18598	unknown
1.546_404.19069	2-[(3R,4S)-1-Benzoyl-3-ethyl-4-piperidinyl]-N-(4-fluorobenzyl) acetam ide	--	unknown

A

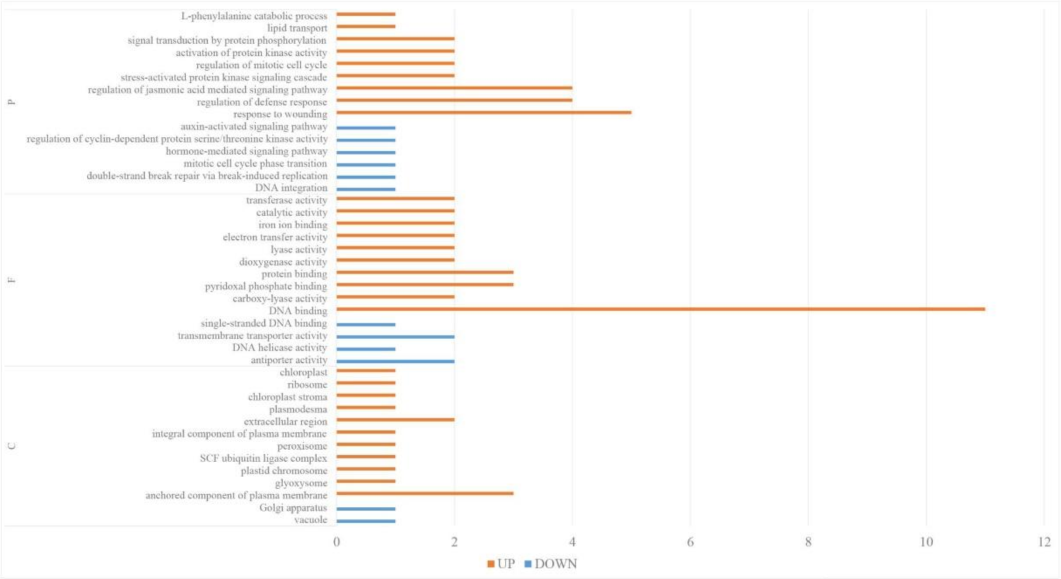

B

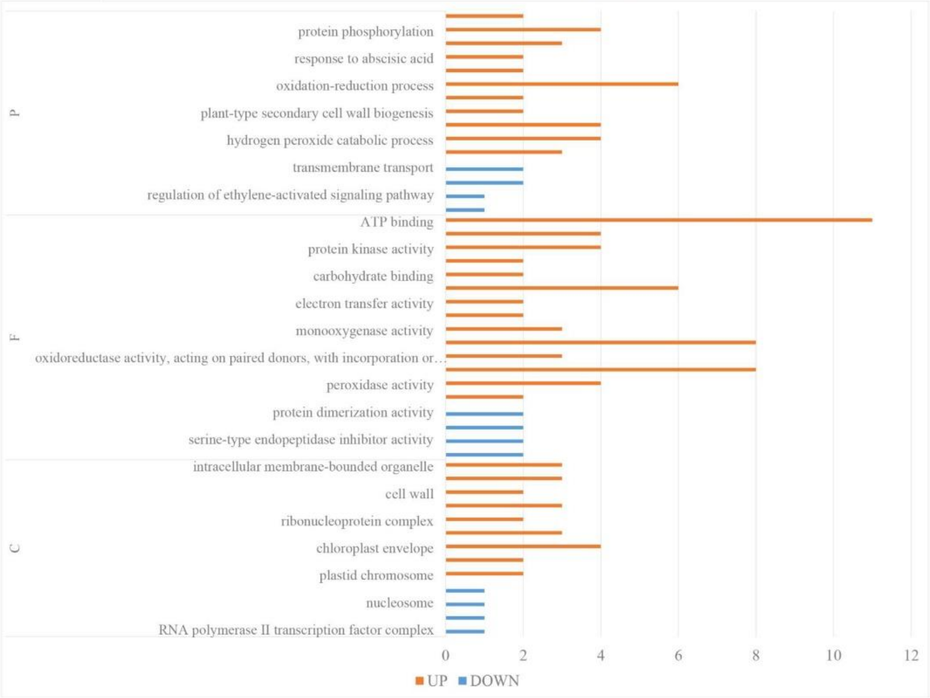

Figure S3. GO enrichment analysis of up- or down-regulated genes in BR-treated rice seedlings. Huanghuazhan: upregulated and downregulated unique GO enrichment classes (A). Chaoyouqianhao: upregulated and downregulated unique GO enrichment classes (B). The x-axis denotes the number of differentiated genes, and the y-axis denotes the GO enrichment annotation.

A

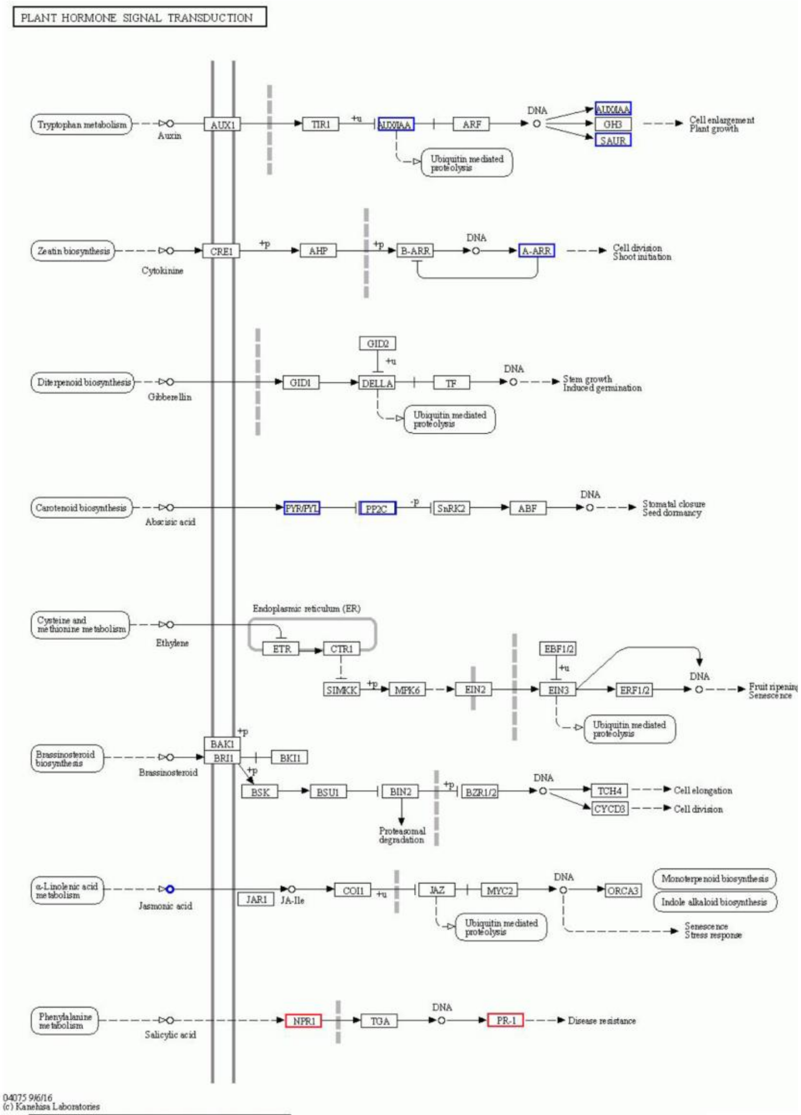
PLANT HORMONE SIGNAL TRANSDUCTION
Tryptophan metabolism
Auxin
AUX1
TIR1
AUX/IAA
ARF
DNA
GH3
SAUR
Cell enlargement
Plant growth
Ubiquitin mediated proteolysis
Zeatin biosynthesis
Cytokinine
CRE1
AHP
B-ARR
A-ARR
Cell division
Shoot initiation
Diterpenoid biosynthesis
Gibberellin
GID1
GID2
DELLA
TF
Stem growth
Induced germination
Carotenoid biosynthesis
Abscisic acid
PYR/PYL
PP2C
SnRK2
ABF
Stomatal closure
Seed dormancy
Cysteine and methionine metabolism
Ethylene
Endoplasmic reticulum (ER)
ETR
CTR1
SIMKK
MPK6
EIN2
EBF1/2
EIN3
ERF1/2
Fruit ripening
Senescence
Brassinosteroid biosynthesis
Brassinosteroid
BAK1
BRI1
BKI1
BSK
BSU1
BIN2
BZR1/2
TCH4
CYCD3
Cell elongation
Cell division
Proteasomal degradation
α-Linolenic acid metabolism
Jasmonic acid
JAR1
JA-Ile
COI1
JAZ
MYC2
ORCA3
Monoterpenoid biosynthesis
Indole alkaloid biosynthesis
Senescence
Stress response
Phenylalanine metabolism
Salicylic acid
NPR1
TGA
PR-1
Disease resistance
04075 9/6/16
(c) Kanehisa Laboratories

B

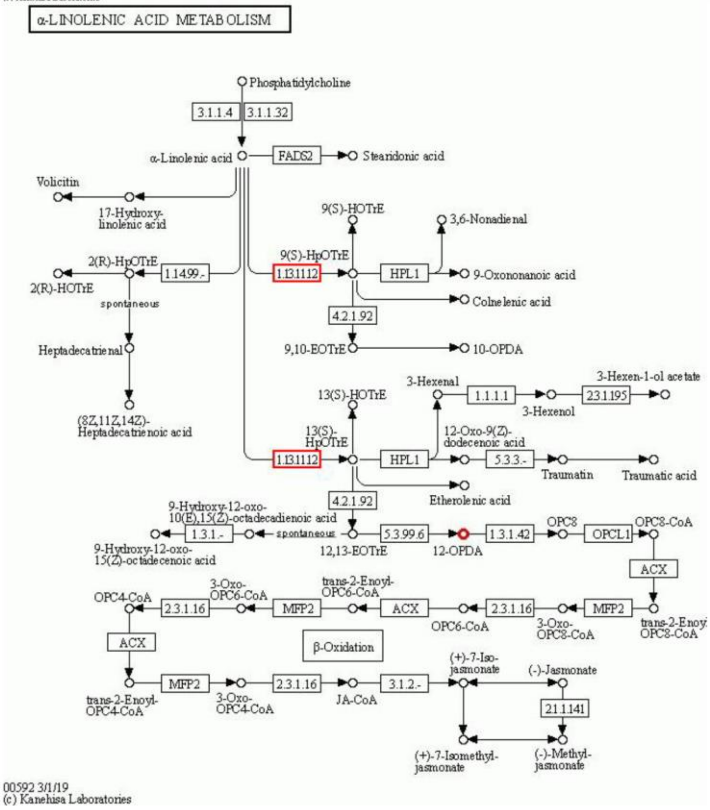
α-LINOLENIC ACID METABOLISM
Phosphatidylcholine
3.1.1.4
3.1.1.32
α-Linolenic acid
FADS2
Stearidonic acid
Volicitin
17-Hydroxy-linolenic acid
9(S)-HOTrE
3,6-Nonadienal
9(S)-HpOTrE
1.13.11.12
HPL1
9-Oxononanoic acid
Colnelenic acid
4.2.1.92
9,10-EOTrE
10-OPDA
2(R)-HpOTrE
2(R)-HOTrE
1.14.99.-
spontaneous
Heptadecatrienal
(8Z,11Z,14Z)-Heptadecatrienoic acid
13(S)-HOTrE
3-Hexenal
1.1.1.1
3-Hexenol
2.3.1.195
3-Hexen-1-ol acetate
13(S)-HpOTrE
12-Oxo-9(Z)-dodecenoic acid
5.3.3.-
Traumatin
Traumatic acid
Etherolenic acid
9-Hydroxy-12-oxo-10(E),15(Z)-octadecadienoic acid
1.3.1.-
9-Hydroxy-12-oxo-15(Z)-octadecenoic acid
12,13-EOTrE
5.3.99.6
12-OPDA
1.3.1.42
OPC8
OPCL1
OPC8-CoA
ACX
trans-2-Enoyl-OPC8-CoA
MFP2
3-Oxo-OPC8-CoA
2.3.1.16
OPC6-CoA
trans-2-Enoyl-OPC6-CoA
3-Oxo-OPC6-CoA
OPC4-CoA
β-Oxidation
trans-2-Enoyl-OPC4-CoA
3-Oxo-OPC4-CoA
JA-CoA
3.1.2.-
(+)-7-Iso-jasmonate
(-)-Jasmonate
2.1.1.141
(+)-7-Isomethyl-jasmonate
(-)-Methyl-jasmonate
00592 3/1/19
(c) Kanehisa Laboratories

Figure S4. Enrichment pathway of genes and metabolites in Huanghuazhan under salt stress mediated by brassinolide. Phytohormone signaling pathway (**A**), α-linolenic acid metabolic pathway (**B**). Red highlighted areas in A and B represent significantly up-regulated gene or metabolite levels, and blue indicates significantly down-regulated genes or metabolite levels with reduced abundance.

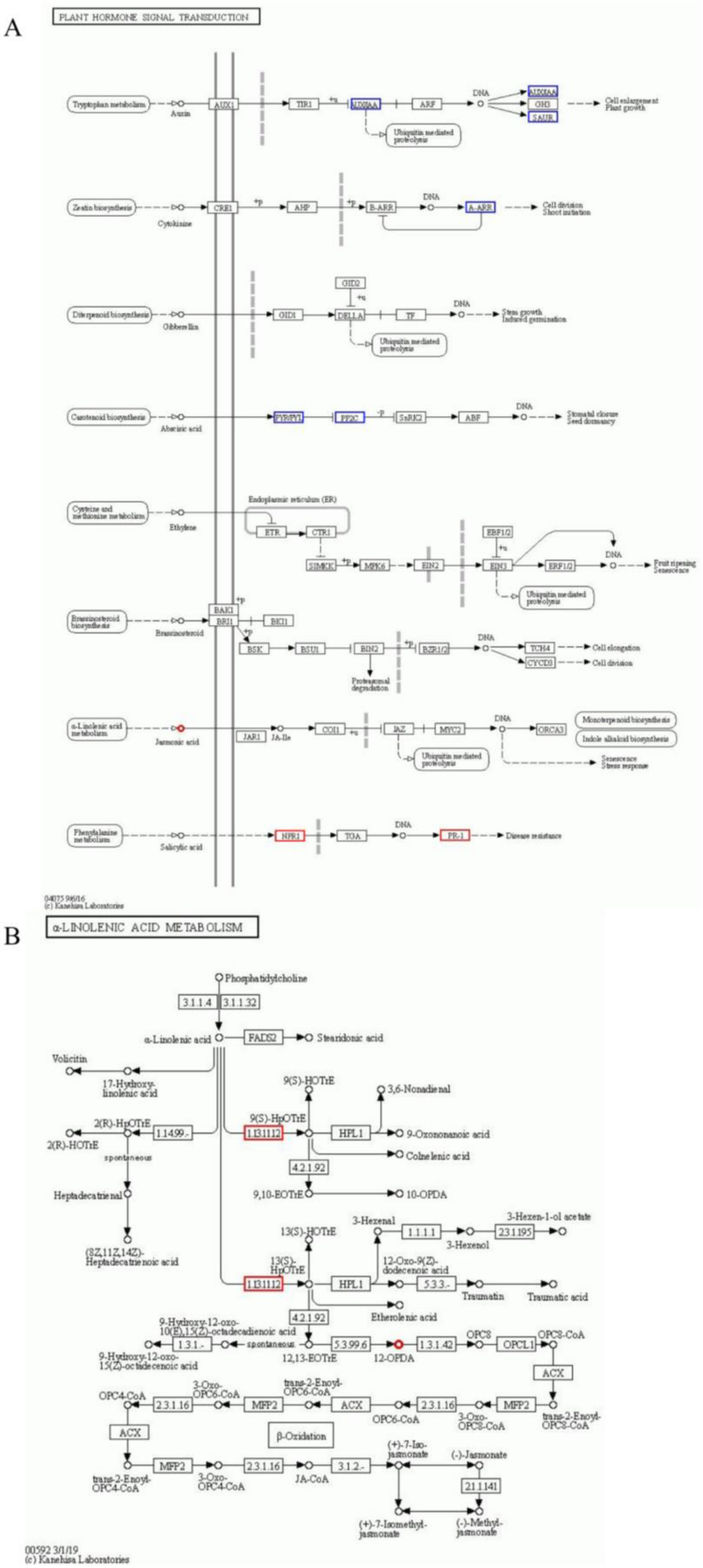

Figure S5. Enrichment pathway of genes and metabolites in Chaoyouqianhao under salt stress mediated by brassinolide. Phytohormone signaling pathway (**A**), α-linolenic acid metabolic pathway (**B**). Red highlighted areas in A and B represent

significantly up-regulated gene or metabolite levels, and blue indicates significantly down-regulated genes or metabolite levels with reduced abundance.
